# P300 Measures and Drive-Related Risks: A Systematic Review and Meta-Analysis

**DOI:** 10.3390/ijerph17155266

**Published:** 2020-07-22

**Authors:** Chao Fang, Yamei Zhang, Mingyi Zhang, Qun Fang

**Affiliations:** 1 Department of Pharmacology, Fourth Military Medical University, Xi’an 710032, China; fang1989@fmmu.edu.cn; 2 College of Architecture and Urban Planning, Qingdao University of Technology, Qingdao 266033, China; zhangyamei@qut.edu.cn; 3 School of Civil Engineering, Qingdao University of Technology, Qingdao 266033, China; zhangmingyi@qut.edu.cn; 4 School of Physical Education, Qingdao University, Qingdao 266071, China

**Keywords:** road safety, drive-related risks, P300, driving performance

## Abstract

Detecting signs for an increased level of risk during driving are critical for the effective prevention of road traffic accidents. The current study searched for literature through major databases such as PubMed, EBSCO, IEEE, and ScienceDirect. A total of 14 articles that measured P300 components in relation to driving tasks were included for a systematic review and meta-analysis. The risk factors investigated in the reviewed articles were summarized in five categories, including reduced attention, distraction, alcohol, challenging situations on the road, and negative emotion. A meta-analysis was conducted at both behavioral and neural levels. Behavioral performance was measured by the reaction time and driving performance, while the neural response was measured by P300 amplitude and latency. A significant increase in reaction time was identified when drivers were exposed to the risk factors. In addition, the significant effects of a reduced P300 amplitude and prolonged P300 latency indicated a reduced capacity for cognitive information processing. There was a tendency of driving performance decrement in relation to the risk factors, however, the effect was non-significant due to considerable variations and heterogeneity across the included studies. The results led to the conclusion that the P300 amplitude and latency are reliable indicators and predictors of the increased risk in driving. Future applications of the P300-based brain–computer interface (BCI) system may make considerable contributions toward preventing road traffic accidents.

## 1. Introduction

Road traffic injuries are now the leading cause of death worldwide for children and young adults aged 5–29 years [1]. According to the statistics provided by the World Health Organization (WHO), the number of annual road traffic deaths has been increasing over the past two decades, from 1.15 million in 2000 to 1.35 million in 2016. In addition, the rate of road traffic deaths per 100,000 has remained above 18% (e.g., 18.8% in 2000 and 18.2% in 2016), with only minimal change over the same period of time. The prevention of road traffic accidents includes a series of critical components such as managing speed, reducing drink-driving, increasing seat-belt use, and increasing child restraint use [1,2]. Despite tremendous endeavors aiming to reduce accidents as well as the corresponding consequences, both the number and rate of road traffic deaths remain unacceptably high, suggesting the limited effect of the current prevention strategies and the need for innovations in traffic risk mitigation.

Previous research has proposed that the technological countermeasure based on brain activity is the most predictive and reliable approach to detect mental states and prevent driver fatigue [3,4]. Continuous recording of the mental state is essential for an adaptive and automated system which can optimally match an individual’s functional capacity with particular tasks [5]. Recently, technical progresses in filtering artifacts and improving the quality of signals enable a large amount of useful information to be derived from brain signals, thus making the application of the neurophysiological measurements feasible in operational environments [6].

One of the promising applications to enhance road traffic safety is the event-related potential (ERP) technique, which allows researchers to assess the risk in driving based on the real-time neural information. Dynamic measures of the ERP components provide a direct insight into drivers’ mental states [7,8]. Compared with traditional psychometric tools such as self-report questionnaires, the ERP technique indicates prominent advantages in monitoring mental states during driving. ERPs reveal brain activities in response to specific events or stimuli [9,10]. The potentials generated in the brain are attributed to the cortical neurons firing in synchrony while processing information [11]. Driving requires substantial cognitive efforts. It is essential for the safety concern that drivers continuously make correct decisions and adjust their driving behaviors based on their evaluation and anticipation of situations on the road. Therefore, the ERP technique is a suitable instrument to measure the mental workload and capacity for efficient information processing while driving [12]. In addition, ERPs are time-locked to the onset of a stimulus. The excellent temporal resolution reveals a precise timing with respect to a driver’s neural responses to an event [13].

The ERP patterns involve the waveform variations in amplitude and latency [10]. While the amplitude accounts for the amount of attentional resources allocated to the task, the latency reflects the speed of cognitive information processing [14]. Among the ERP components, P300 has been widely used to assess cognitive function [15]. It is the positive peak amplitude within a time window between 250 and 500 ms [16]. A greater P300 amplitude and shorter latency are associated with a superior performance in cognitive information processing [16,17]. In contrast, a reduced amplitude or prolonged latency is often observed in distraction [18,19], mental fatigue [19,20], and mind wandering [21,22], suggesting a reduced capacity and efficiency of cognitive information processing. The risk of conducting unsafe driving behaviors and making operation errors increases when drivers experience a non-optimal mental state [23,24]. Therefore, the predictive value of P300 on mental state and cognitive performance makes it a potential indicator in the practice of preventing road traffic accidents.

With the accumulating evidence from the research, which measured P300 components in the driving task, researchers have gained in-depth knowledge about P300 variations in relation to the increased level of risk during driving. For example, prolonged driving has been found to cause mental fatigue and reduce drivers’ attention on the task, which can be reflected in the P300 amplitude and latency. It is our interest to find out whether the changes at the neural level are consistent across the studies so that the presence of specific P300 patterns can imply certain mental states and cognitive functions. To answer the primary research question, the current study conducted a systematic review on the drive-related risks investigated in the included studies. In addition, a meta-analysis was performed to provide quantitative information on the behavioral performance and P300 measures. The results will lead to a discussion on whether P300 can be used as a reliable indicator to assess and predict the level of risk during driving. The application of a P300-based system to detect signs of an increased risk would make considerable contributions to the prevention of road traffic accidents.

## 2. Materials and Methods

### 2.1. Search Strategy

Literature was searched through PubMed, EBSCO, IEEE, and ScienceDirect. Due to the interdisciplinary feature of the current study, major databases in neuroscience, health, and engineering were chosen for literature search. While PubMed was selected as a bench-mark database for neuroscience and health, IEEE provided a specific focus on publications related to engineering and computer science. To identify the studies which applied P300 measures to examine neural responses to an increased level of risk in driving, the following key terms were used for the literature search: “road safety OR road accident OR driving risk OR driving behavior OR distracted driving” AND “ERP OR P300 OR P3”. The initial search results were screened based on the title and abstract. Then the full-text examination was conducted on the remaining articles to determine the eligibility for inclusion. To ensure a thorough search for eligible articles, a snowball search was conducted in addition to the database search. While performing the full-text examination, the authors recorded the references that may fit into the focus of the current review. Then the full-text examination was conducted on the recorded references to evaluate the eligibility for inclusion. Two authors (C.F. and Y.Z.) worked independently on the literature search and selection. Disagreements were discussed with the other authors in consensus meetings.

### 2.2. Inclusion Criteria

The current review is focused on the ERP studies that investigated driving behaviors and neural activation patterns in response to an increased risk in driving. To satisfy the research purpose, eligible literatures should meet the following criteria: (1) peer-reviewed articles or conference proceeding papers written in English; (2) P300 amplitude or latency was the measure of neural activation; (3) the experiment was conducted in the context of driving, either in a simulator or on the road; (4) the experimental design should assess changes in P300 components or behavioral performance by comparing the control group with the experimental group, or comparing the baseline state with the state at the end of the experiment. The evaluation of the literature eligibility was conducted in accordance with the predetermined criteria. Failure to meet one of the criteria would result in exclusion of the article.

### 2.3. Data Extraction and Synthesis

Due to the research focus being on the risk-induced responses at both neural and behavioral levels, the information and data with respect to risk factors, P300 components, and performance measures were extracted. The risk factors summarized drive-related risks (e.g., mental fatigue and distraction) in the studies. It is our main interest to know how these risk factors were manipulated in each experiment. P300 components were considered the indicators of neural responses to an increased level of risk in driving. The amplitude and latency measure different aspects of cognitive function, with the amplitude reflecting the amount of cortical activity and the latency indicating the speed of information processing [10,25]. Therefore, quantitative results regarding the P300 measures were synthesized across the included studies.

The performance measures were retrieved from the oddball paradigm and driving task. The oddball paradigm is a commonly used design in ERP studies which requires subjects to discriminate the frequent stimuli (target stimuli) from the infrequent stimuli (background stimuli) and respond to the target stimuli by counting or pressing a button [25,26]. Thus, reaction time is a primary measure of the subjects’ performances in the task. In addition to the oddball paradigm, a driving task provides a variety of performance measures such as mean speed, speed variability, lane deviation, and driving errors [27,28]. Because reaction time and driving behaviors are essential to driving safety, the current review conducted separate statistical analyses on the reaction time of the oddball paradigm and performance of the driving task.

### 2.4. Statistical Analysis

The statistical analysis was conducted by Comprehensive Meta-Analysis, Version 3 (Biostat, Inc., Englewood, NJ, USA). Hedges’ *g* was considered a conservative estimate for a small sample size [29]. The effect size was calculated based on the mean, sample size, and *p* value. The magnitudes of the effect size were categorized as small (Hedges’ *g* = 0.2–0.5), medium (Hedges’ *g* = 0.5–0.8), and large (Hedges’ *g* > 0.8). A random-effects model was used for the consideration of heterogeneity across the individual studies [30]. The Q test was performed to measure heterogeneity, with *I*^2^ < 25% for likely heterogeneity, 25% < *I*^2^ < 50% for moderate heterogeneity, and *I*^2^ > 50% for considerable heterogeneity [31]. The Egger’s regression was used as a measure of publication bias. A *p*-value of 0.05 was the cut-off point for significant results.

## 3. Results

### 3.1. Study Selection

An initial search through the databases identified a total of 515 articles. The first phase screened 392 articles based on title and abstract examination. Then, the eligibility of the remaining articles (*N* = 123) was determined by a full-text examination. There were 110 articles excluded in this phase because of the following reasons: duplicates (*N* = 32), not using P300 components (*N* = 36), conference abstract (*N* = 17), and no driving task (*N* = 25). One study was included by means of snowball search [32]. The two-phase screening process resulted in 14 articles that met the inclusion criteria of the current systematic review and meta-analysis.

### 3.2. Summary of the Risk Factors in the Driving Tasks

A summary of the study characteristics is listed in Table 1. The risk factors involved in the included articles can be summarized in five categories, including distracted driving (*N* = 5) [12,27,32,33,34], reduced attention (*N* = 5) [19,35,36,37,38], increased difficulty in driving (*N* = 2) [27,28], alcohol (*N* = 2) [32,39], and negative emotion (*N* = 2) [40,41]. Distracted driving is a major cause of traffic accidents, which is related to driving behaviors such as using a cell phone and talking to passengers while driving. A common approach to imitate distracted driving was to integrate the oddball paradigm into the driving task [32,33,34]. Therefore, attentional resources were divided from the primary driving task as subjects tried to identify the target stimuli from the background stimuli.

A reduced attention was observed in a variety of conditions such as mental fatigue, monotonous routes, and automated driving. Mental fatigue was induced by prolonged driving as subjects continuously drove for approximately 2 to 4 h [19,35]. In the study regarding monotonous driving, subjects completed two simulated drives each day for five consecutive days [37]. To imitate the monotonous nature of daily driving, the scenarios were identical across all simulated drives. A new research focus lies in automated driving which has been found to influence a driver’s attention on task [38]. Subjects were told that the automated car was not always reliable so that they may need to manually control the car occasionally. During the automated driving mode, the subjects’ attentional states were recorded.

There were two studies on the impact of task difficulty on driving performance and neural activation. A direct approach to increase the difficulty of the simulated driving was to change the road conditions from a road with slight bends to one with sharp curves [27]. The number of vehicles on the road was another variable researchers used to manipulate the task difficulty. Driving on a busy road requires drivers to process greater amount of information, thus increasing drivers’ mental workload. Another study examined the influence of the task difficulty by exposing novice drivers and experienced drivers to the same scenarios [28]. Compared with the experienced drivers, the novice drivers recruited more mental resources to process the information on the road, suggesting a higher level of mental workload in the novice drivers.

Drinking has been a major cause for road traffic accidents. Two of the included studies investigated the effects of alcohol on a driver’s performance from different perspectives. Ebe and colleagues designed a study focusing on the effects of drinking a small dose of alcohol on driving [39]. Subjects participated in two driving tests, with and without consuming alcohol. In the test under the alcohol condition, subjects consumed a can of beer (350 mL, 5% alcohol) in 12 min. In the test on the other day, subjects drank the same amount of water in 12 min. Driving performance as well as ERP measures were compared between the alcohol condition and the normal condition. The other included study had a specific emphasis on driving performance at different blood alcohol concentration levels [32]. Subjects consumed a 250 mL beverage with different alcohol dosages 5 min prior to each driving test. A total of five tests were performed at the blood alcohol concentration levels of 0.00% (placebo), 0.02%, 0.05%, 0.08%, and 0.10%. By using such treatments to control alcohol consumption, researchers were able to investigate the effects of different dosages on subjects’ driving performance.

Angry driving has received an increasing attention from researchers as it has become a real concern in our daily lives [42]. Anger tends to raise an individuals’ arousal level which can lead to aggressive and risky behaviors [43]. One of the included studies stimulated anger by asking subjects to recall and write down personal experiences that made them angry [40]. A follow-up questionnaire, the Brief Mood Introspection Scale (BMIS), was completed to assess the subjects’ anger levels. The other study induced anger by means of an anger elicitation gambling task (AEGT) [41]. The researchers designed a two-choice task for 20 trials. Subjects were told they had a chance to win, but the game was set so the subjects kept losing over the 20 trials. The anger level was believed to raise due to the continuous loss. After the gambling task, researchers assessed if subjects were qualified to conduct the following driving test based on subjects’ self-reported anger scores.

### 3.3. Meta-Analysis on Behavioral Measures

#### 3.3.1. Reaction Time

**Figure 1:**
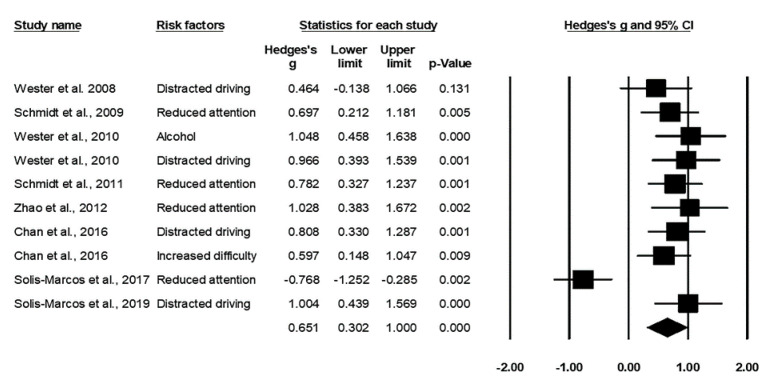
Forest plot for reaction times in relation to the risk factors.

A positive value of the effect size represented an increased reaction time in response to the risk factors, whereas a negative effect size indicated a decreased reaction time. The results indicated a medium effect size, Hedges’ *g* = 0.651, *p* < 0.001, and the positive value suggests that the risk factors such as distracted driving, reduced attention, increased task difficulty, and alcohol consumption significantly prolonged the reaction time of the oddball paradigm. The result of heterogeneity test was non-significant, Q_9_ = 4.16, *I*^2^ =7.42%, *p* = 0.91, suggesting small variations across the studies. The Egger’s regression test was non-significant, *t*_8_ = 0.99, *p* = 0.35, indicating no publication bias in the studies. The forest plot for reaction times is presented in Figure 1.

#### 3.3.2. Driving Performance

There were 10 studies which provided 18 measures of driving performance in relation to an increased risk level. The risk factors that were investigated in the studies involved distracted driving [27,32,33], increased task difficulty in driving [27,28], reduced attention [19,37,38], alcohol [32,39], and negative emotion [40,44]. These risk factors influenced the subjects’ performances regarding a variety of measures such as driving error, risky behaviors, collisions, central line crossing, lane deviation, lateral variability, and speed variability.

The driving error is the mistakes subjects made during the driving task, such as not following the predefined path and turning at a wrong intersection [28]. The frequencies of risky behaviors, collisions, and central line crossing were counted during the task, with greater frequencies indicating worse performances [28,44]. The lane deviation was calculated by the standard deviation of the distance from the center of the lane [32,33,37,40]. A larger deviation was interpreted as a worse driving performance. The lateral variability was defined as the standard deviation of the car’s lateral position [37]. A small lateral variability suggests a relatively straight path which was considered a better performance as opposed to a large lateral variability. The speed variability reflects the smoothness of driving, with a small speed variability indicating better performance [37].

**Figure 2:**
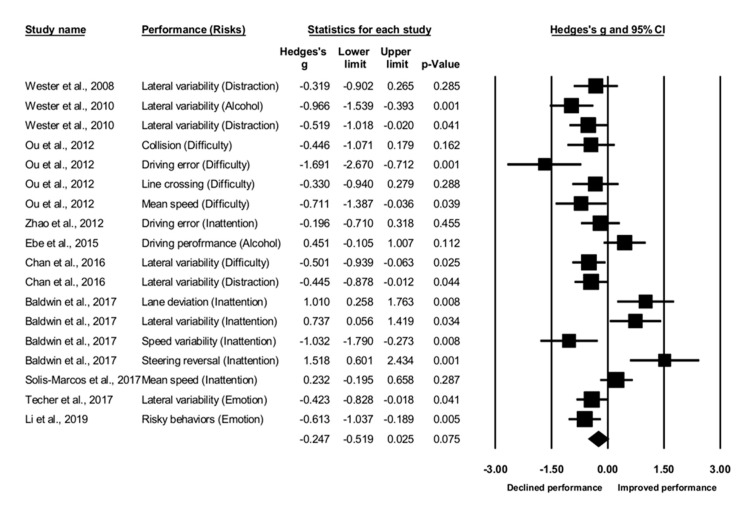
Forest plot for driving performance in relation to the risk factors.

The meta-analysis indicated a negative, but non-significant effect size in terms of driving performance (Figure 2), Hedges’ *g* = −0.247, *p* = 0.075. The risk factors resulted in a decline in driving performance, but the change did not reach the significant level. A heterogeneity test indicated significant variations across the studies, Q_17_ = 71.28, *I*^2^ = 76.09%, *p* < 0.001. The significant heterogeneity was likely a result of the diverse measures of driving performance. There was no publication bias as the Egger’s regression test was non-significant, *t*_16_ = 0.66, *p* = 0.52. In sum, the result indicated a negative, non-significant effect size. However, it is necessary to interpret the result with caution due to the significant heterogeneity across the included studies.

### 3.4. Meta-Analysis on P300 Variations

#### 3.4.1. Amplitude

**Figure 3:**
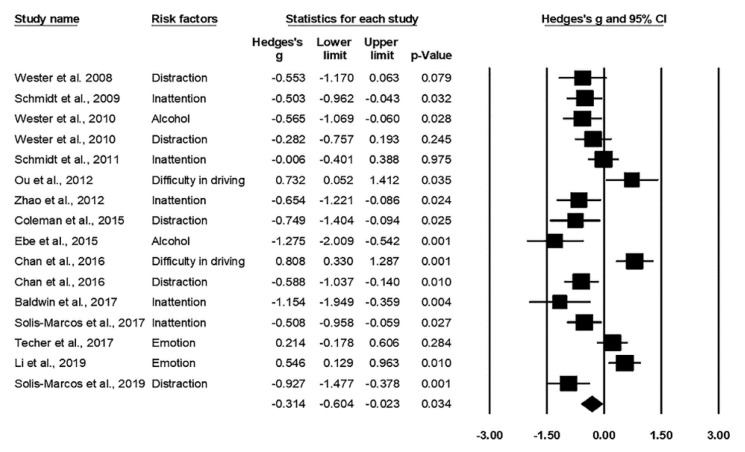
Forest plot for P300 amplitude in relation to the risk factors.

The significant effect size indicated a reduced P300 amplitude when subjects were exposed to risk factors while driving (Figure 3), Hedges’ *g* = −0.314, *p* = 0.034. The reduced amplitude was interpreted as a smaller amount of attentional resources allocated to the primary driving task, especially in the situations of multiple tasks [12,27,32,33,34], reduced attention due to the influence of alcohol [32,39], monotonous driving [37,38], and mental fatigue [19,35,36]. A likely heterogeneity was identified according to *I*^2^, but the *p*-value indicated a non-significant result, Q_17_ = 21.02, *I*^2^ = 20.64%, *p* = 0.14. Egger’s regression suggests no publication bias in the studies, *t*_14_ = 2.03, *p* = 0.06.

#### 3.4.2. Latency

**Figure 4:**
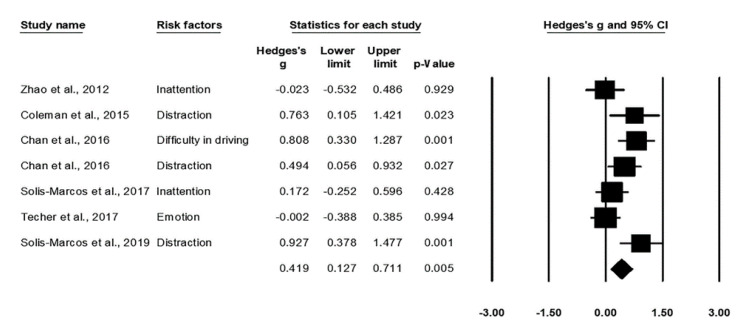
Forest plot for P300 latency in relation to the risk factors.

The effect size indicated a significant delay in P300 latency in relation to the risk factors such as reduced attention [19,38], distracted driving [12,27,34], increased difficulty in the driving task [27], and negative emotion [40], Hedges’ *g* = 0.419, *p* = 0.005. The prolonged latency is associated with a declined capacity in terms of information processing. Subjects may take longer time to make decisions and take actions in the face of an emergent situation on the road. The heterogeneity across the studies was non-significant, Q_6_ = 7.88, *I*^2^ = 23.90%, *p* = 0.25, suggesting a limited impact on the result. According to the Egger’s regression, no significant publication bias was identified, *t*_5_ = 1.73, *p* = 0.14. Figure 4 displays the forest plot for P300 latency.

## 4. Discussion

### 4.1. Summary of the Main Findings

The current review included 14 studies which applied the ERP technique to driving tasks. Subjects were exposed to drive-related risks such as a reduced attention, distraction, alcohol, challenging situations on the road, and negative emotion. Therefore, the included studies provided a comprehensive examination on common risk factors for road traffic accidents. We conducted meta-analyses on four aspects to investigate the influences of the risk factors on drivers’ behavioral performances and neural activation. Specifically, the behavioral performance was measured by the reaction time of the oddball test and driving performance. In addition, P300 amplitude and latency reflected the neural responses to the drive-related risks. The results indicated an overall decline in the capacity for information processing, evidenced by a prolonged reaction time, reduced P300 amplitude, and delayed P300 latency. As for the driving performance negative, the effect size suggested a tendency of performance decrement associated with the increased risk level, but the result was non-significant, and the heterogeneity test indicated considerable variations with respect to the performance measures across the studies.

### 4.2. P300 Components—Predictors for Driving Behaviors

Whereas a significant effect was identified at the neural level, there was no significant change in the driving performance. The results suggest that driving performance may not be as sensitive to the increased risk as the P300 components. Good evidence can be found in the research on the impact of “one drink” of alcohol on drivers’ performances [39]. In this simulated driving experiment, subjects consumed a can of beer (350 mL, 5% alcohol by volume) before the experiment. Such an amount of alcohol consumption did not cause a performance decline in the driving task. However, neural evidence indicated a significant reduction in the P300 amplitude which was the evidence for less functional capacity in terms of information processing. Therefore, it is likely that risk factors, such as alcohol consumption, may affect neural processes for driving even at a level which is too low to modify behavior [39].

A current neural model provides the theoretical foundation to explain the relationship between behavioral performance and neural activation. The model of facilitation system proposes that a compensatory mechanism can be activated to compensate for the declined cognitive function due to an increased mental workload or accumulated fatigue [45,46]. Research has shown that an additional neural circuit was recruited, known as overactivation, to maintain performance in fatigue-inducing tasks [46,47]. Therefore, signs for the performance decrement may be first observed in neural activation. The compensatory mechanism emphasizes the importance of using neural evidence to detect the situation in which functional decline only occurs at the neural level rather than in the behavioral performance. Theoretically, there should be a time interval during which the increased risk has not resulted in performance decline. Taking advantage of this window would effectively reduce road traffic accidents.

According to the results of the meta-analysis, variations of P300 components in relation to the increased drive-related risk are characterized by a reduced amplitude and prolonged latency. The existence of such variations could be considered indicative of a reduced capacity for optimal cognitive function [14,16]. If the brain activity is being monitored by a P300-based brain–computer interface (BCI) system, the system can raise a driver’s awareness of the increased risk level by means of certain behavioral interventions [48].

Previous research has shown that drivers, especially the young drivers, tend to be overconfident and thus underestimate potential risks [49,50]. In an experiment for distracted driving, researchers compared participants’ subjective estimates of performance decrement with their actual performance decrement [51]. The results indicated a greater decrement in the actual performance and a smaller estimate of distraction, which implied that the participants underestimated the detrimental influence of distraction on their driving performance. Consistent findings were identified in one of the included studies which compared self-reported vigilance with the vigilance state assessed by a set of physiological and performance measures [35]. The subjects reported an improved vigilance in the last 20 min of the 3 h driving task. However, the subjective assessment was contrary to the continued decline in vigilance states assessed by the physiological and performance measures. Given the research evidence that drivers are likely to misjudge their abilities to address an emergent situation, a real-time monitoring system is helpful to avoid traffic accidents due to the underestimation of risk while driving.

### 4.3. Application of P300 Research to Reduce Road Traffic Accidents

The significant findings at the neural level suggest that the P300 amplitude and latency can be used as valid and reliable indicators and predictors of drivers’ mental states as well as cognitive performance. It has been known that a reduced P300 amplitude is a sign of decreased neural activity and the prolonged latency indicates delayed cognitive information processing [52]. Based on the findings of the current systematic review and previous P300 research, a BCI system can be designed to predict drivers’ performances and assess the level of risk according to the characteristics of P300 components. When the reduced P300 amplitude and prolonged latency are identified, an alertness can be sent so that drivers are able to notice the increased risk and potential performance decrement. Therefore, a practical solution to reduce road traffic accidents is to apply a P300-based BCI system to realistic driving. In fact, the system has been applied to the clinical diagnosis of neurological impairments such as strokes [44,53,54], autism [55,56], and Parkinson’s disease [57,58]. Recently, research on cognitive practices such as spelling [59,60], counting [61], and random dot motion [15] also reported encouraging results. The research progresses in P300-based BCI systems make it feasible to assist drivers in preventing road traffic accidents.

Another contribution of P300 research is to provide neural information on the effectiveness of a warning system in preventing road traffic accidents. A common experimental design requires subjects to respond to the random decelerations of a leading vehicle [62,63,64]. Research has indicated that auditory warnings given before the deceleration of the leading vehicle significantly reduced subjects’ reaction times to the stimulus [62,63]. In addition to the benefits to behavioral performance, neural signals also suggested positive changes. A smaller P300 amplitude was considered evidence that fewer attentional resources were needed in the driving task, while a reduced P300 latency indicated a reduced time for cognitive information processing. The evidence at the neural levels supported the effectiveness of the auditory warning system in avoiding rear-end collisions. More importantly, these studies justified using P300 components to assess novel approaches to reduce road traffic accidents.

## 5. Limitations and Future Direction

The current review included a total of 14 studies which covered a variety of risk factors for road traffic accidents. In addition to the common risks, such as distracted driving and reduced attention on task, a few studies examined the effects of alcohol [32,39] and negative emotion on driving performance [40,44]. The broad category of drive-related risk could be considered a merit of the current research, but it also brought up a reasonable concern with powerful evidence because of the relatively small sample size for each risk factor. Therefore, subsequent studies on this topic are warranted.

Although this review focused on P300 components, it is still necessary to point out that other ERP components can be taken into account as measures of driving performance. For example, N1 is an initial deflection peaking around 100 ms to 150 ms [14,65]. This component is an important measure for perception and attention. In addition, the lambda response is another ERP measure related to the positive peak around 100 ms (P1 component). This measure is a reliable indicator of visual information processing, and thus has raised researchers’ interest in applying the lambda response to the research on driving performance [66,67]. Due to the limited number of studies with respect to N1 or the lambda response, only P300 components were analyzed in the current review, but it is important to realize that an increased number of studies in the ERP measures other than P300 are needed.

## 6. Conclusions

This review investigated both neural and behavioral changes when drivers were exposed to risk factors while driving. Performance decrement was evidenced by a significant increase in the reaction time of the oddball paradigm, and a declined capacity for cognitive function was identified in the reduced P300 amplitude and prolonged P300 latency. The findings at both behavioral and neural levels suggest a promising solution to reduce road traffic accidents. By tracking the real-time neural information while driving, a P300-based BCI system is feasible to monitor drivers’ risk levels and thus help to prevent road traffic accidents.

## Figures and Tables

**Table 1:** Characteristics of the included studies.

Study	Subjects	Risk Factors	P300 Components	Performance Measures
Wester et al., 2008 [33]	*N* = 20 Age: 23.1	Distracted driving	Amplitude (−)	Oddball test: reaction time (n.s.) Lane deviation (n.s.)
Schmidt et al., 2009 [35]	*N* = 19 Age: 29.4	Reduced attention—metal fatigue	Amplitude (−)	Oddball test: reaction time (+)
Wester et al., 2010 [32]	*N* = 32 Age: 23.5	Alcohol and distracted driving	Alcohol: Amplitude (−) Distracted driving: Amplitude (n.s.)	Alcohol: Steering error (+) Oddball test: reaction time (+) Distracted driving: Lane deviation (+)
Schmidt et al., 2011 [36]	*N* = 20 Age: 26.6	Reduced attention—metal fatigue	Amplitude (n.s.)	Oddball test: reaction time (+)
Ou et al., 2012 [28]	*N* = 17 Age: 21.9	Increased difficulty in driving	Amplitude (+)	Number of wrong turns (+) Mean speed (−) Central line crossing (+) Frequency of collision (+)
Zhao et al., 2012 [19]	*N* = 13 Age: 25.8	Reduced attention—metal fatigue	Amplitude (−) Latency (n.s.)	Oddball test: reaction time (+)
Coleman et al., 2015 [34]	*N* = 10 Age: 24.7	Distracted driving	Amplitude (−) Latency (+)	Not reported oddball test results.
Ebe et al., 2015 [39]	*N* = 12 Age: 21–35	Alcohol	Amplitude (−)	Lane deviation (n.s.) Distance headway (n.s.) Response time (n.s.)
Chan et al., 2016 [27]	*N* = 27 Age: 20	Increased difficulty and distracted driving	Amplitude (−) Latency (+)	Mean speed (−) Lane deviation (+) Oddball test: reaction time (+)
Baldwin et al., 2017 [37]	*N* = 9 Age: 24	Reduced attention—automated driving	Amplitude (−)	Speed variability (+) Lane deviation (−) Lateral position variability (−) Steering reversal (−)
Solis-Marcos et al., 2017 [38]	*N* = 20 Age: 27.1	Reduced attention—monotonous driving	Amplitude (−) Latency (n.s.)	Oddball test: reaction time (n.s.)
Techer et al., 2017 [40]	*N* = 33 Age: 32.3	Negative emotion: anger	Amplitude (n.s.) Latency (n.s.)	Response time (n.s.) Distance headway (n.s.) Lane deviation (n.s.) Lateral position variability (+)
Solis-Marcos and Kircher, 2018 [12]	*N* = 17 Age: 23.2	Distracted driving	Amplitude (−) Latency (+)	Oddball test: reaction time (−)
Li et al., 2019 [41]	*N* = 28 Age: 20.8	Negative emotion: anger	Amplitude (+)	Risky driving behaviors (+)

Note: n.s., non-significant result; (−), decrease; (+) increase.
